# Comparison of Parental and Children’s Dental Anxiety Levels Using the Modified Dental Anxiety Scale and Modified Short State-Trait Anxiety Inventory (EMOJI) Scale

**DOI:** 10.3390/children11121532

**Published:** 2024-12-18

**Authors:** Abdulfatah AlAzmah, Rajashekhara Bhari Sharanesha, AlWaleed Abushanan, Abdullah bahjat Khojah, Alhussain ali Dhaafi, Abdulaziz Ahmed Almakenzi, Adel S. Alqarni, Maram Alagla, Abdulhamid Al Ghwainem, Sara Alghamdi

**Affiliations:** Department of Pediatric Dentistry, College of Dentistry, Prince Sattam bin Abdulaziz University, Al-Kharj 11942, Saudi Arabia; a.abushanan@psau.edu.sa (A.A.); a.a1.b.khojah@gmail.com (A.b.K.); a.aldaafi1998@gmail.com (A.a.D.); abdulazizam80@yahoo.com (A.A.A.); as.alqarni@psau.edu.sa (A.S.A.); m.alagla@psau.edu.sa (M.A.); a.alghwainem@psau.edu.sa (A.A.G.); sar.alghamdi@psau.edu.sa (S.A.)

**Keywords:** behavior, dental anxiety, Modified Dental Anxiety Scale, short State-Trait Anxiety Inventory scale

## Abstract

Background: The study aimed to assess dental anxiety (self and proxy reported) using a Modified Dental Anxiety Scale (MDAS) and modified short State-Trait Anxiety Inventory scale (Emoji). Methods: 200 children visiting the pediatric dental clinic at the College of Dentistry at Prince Sattam bin Abdulaziz University were recruited to assess their anxiety levels. The anxiety score was measured before and after the treatment using a short STAI scale and MDAS of 200 children using a Modified Dental Anxiety Scale (MDAS) and a modified short STAI (Emoji) Scale. Results: The perception of children with MDAS was found to have the highest mean score of 14.54 ± 3.82 before the dental procedure compared to the mean score of 9.40 ± 2.90 after the dental procedure. This difference was statistically significant (*p* < 0.001). A statistically significant difference was observed in MDAS after the dental procedure and, in short, STAI before the dental procedure. Conclusion: All children improved their dental anxiety levels before and after the procedure. Therefore, procedural experience may improve the child’s response.

## 1. Introduction

Dental fear, a type of anxiety, is a psychological state that often leads to a perpetuating pattern of avoidance or hesitation in seeking dental care, resulting in a negative experience during dental visits [1]. It frequently comes before the reliable challenge of disturbing stimuli, which can occasionally be difficult to recognize [2]. For many children, visiting a dentist is a fearful and stressful experience.

According to various studies, 5–20% of children experience the fear of visiting the dentist [3,4]; this fear of dental procedures results in delaying dental treatment, further deteriorating children’s oral health [5,6]. The fear of going to the dentist and the difficulty in obtaining regular dental care because of dental anxiety is a prevalent issue that ranks fifth among the most feared experiences for people [7]. Dental anxiety among children is a significant barrier to effective patient management. It can lead to disruptive behavior during treatment and may result in dental avoidance later in life, contributing to poor oral health outcomes in adulthood [8].

A pediatric dentist is a specialist who has the ability and the techniques required to manage a child with anxiety and assist children in becoming more lenient toward accepting dental care. Thus, assessing dental anxiety in children is essential not only for providing high-quality clinical care but also for understanding their anxiety levels prior to treatment and the contributing factors. This allows dentists to pinpoint anxious children, leading to better anxiety management and positively influencing their perceptions of dentists and dental procedure appointments. As a result, behavioral science plays a crucial role in dentistry, focusing on and measuring patients’ behaviors toward dental treatment.

Single and multiple-item self-report questionnaires are available to assess dental anxiety. Some widely used multi-item scales include Corah’s Dental Anxiety Scale (CDAS) [9], the Modified Dental Anxiety Scale (MDAS) [10], and the Spielberger State-Trait Anxiety Inventory [11]. Spielberger also created the State-Trait Anxiety Inventory for Children State form (STAIC-S), which consists of 20 items and demonstrates high reliability and satisfactory validity [11]. An anxiety scale is considered ideal when it combines various factors such as ease of clinical use, time efficiency, and applicability in young children with minimal cognitive and linguistic skills. In Japanese, emoji means e(picture)+moji(character). The shortened version of STAI consists of six statements. Scores for this condensed version range from 6 to 24 points, where 6 indicates no anxiety and 24 reflects the highest level of anxiety [12]. The Emojis method was used with the short STAI to more effectively align with younger children’s cognitive and communicative abilities and preferences, allowing them to convey their anxiety independently.

Self-reported survey data often comes from adults, such as parents or referring dentists, instead of being collected directly from children themselves. This approach may not accurately represent the children’s experiences, as it relies heavily on others’ perceptions and predictions [13]. Children are regarded as capable of expressing their anxieties through questionnaires starting at the age of five; therefore, healthcare professionals typically rely on parents to provide precise details about their children’s dental health anxiety [14,15]. Research conducted in the UK, USA, and Europe has evaluated the link between children’s self-reported dental anxiety and the reports provided by their parents or carers, showing a low correlation that tends to favor the children’s assessments [16,17]. In Saudi Arabia, there appears to be a lack of studies investigating the relationship between children’s self-reported anxiety levels and the anxiety levels reported by their parents regarding dental situations involving their children.

This study assessed dental anxiety among children and parents using the Modified Dental Anxiety Scale and modified short STAI (Emoji) scale.

## 2. Materials and Methods

The research proceeded after receiving approval from the Institutional Review Board at Prince Sattam bin Abdulaziz University (Approval No-SCBR-017-2023). Two hundred children aged 6 to 9 years, who visited the pediatric dental clinics from March 2023 to October 2023 at the College of Dentistry, Prince Sattam bin Abdulaziz University, were randomly selected for this study. The sample size needed was calculated to detect a simple correlation of at least *r* = 0.2 using a two-sided test with a significance level of 5% (α = 0.05) and a power of 80% (*β* = 0.2), resulting in an approximate requirement of 194, which was rounded up to 200 participants. Only healthy children whose parents provided informed consent and who themselves gave permission to participate were included. Participants with previous dental experiences were excluded, and emphasis was placed on individuals attending for preventive dental care.

The current study utilized two anxiety assessment tools: the Modified Dental Anxiety Scale (MDAS) and a revised brief State-Trait Anxiety Inventory (STAI). The MDAS consists of five questions (see Table 1). Each question offers five response options, with scores ranging from 1 (not anxious) to 5 (extremely worried) assigned to each option. The scores from the five questions were pooled to obtain an overall dental anxiety score. A score of 19 or higher indicates extreme dental anxiety, a score between 12 and 19 suggests mild dental anxiety, and a score ranging from 5 to 11 denotes no anxiety [18].

A revised short State-Trait Anxiety Inventory (STAI) by Stefan Nilsson et al. involved converting six statements (tenseness, sadness, fear, calmness, excitement, and happiness) into six emoji faces for use in a study with children. The researchers selected these images to align with the emotions expressed in the original short STAI (Table 1). Three emoji faces express negative emotions, specifically tenseness, sadness, and fear, while the other three exhibit positive emotions, representing calmness, excitement, and happiness. Children can choose from these six emoji faces on an electronic device. This modified and user-friendly approach aims to capture individual feelings and preferences. The children were requested to express their feelings before and after the dental procedure. Each item reflecting emotional states and traits was assessed using a 4-point scale for negative emoji faces. On this scale, a score of 1 indicates “not at all”, 2 means “somewhat”, 3 represents “moderately”, and 4 stands for “very much”. For positive emotions, the scoring is reversed: 1 indicates “very much”, 2 means “moderately”, 3 represents “somewhat”, and 4 denotes “not at all”. The child is shown facial expression emojis one at a time and asked to choose each based on their preference. Afterward, the tool calculates a score that reflects the child’s level of anxiety. The scoring range for this tool is between 6 and 24 points, where 6 points indicate no anxiety and 24 points represent the highest level of anxiety. A single researcher documented and analyzed all facial expressions. Additionally, the parents (mothers) of all participating children evaluated their children’s anxiety levels before and after the dental procedure using the Modified Dental Anxiety Scale and a modified short State-Trait Anxiety Inventory (STAI), employing an emoji method.

Anxiety scores were assessed both before and after treatment, using a paired *t*-test for comparison. Additionally, Pearson’s correlation analysis was performed to examine the relationship between proxy scores and the children’s self-reported anxiety levels. The data were processed using appropriate statistical methods through SPSS (IBM Corp. Released 2013. IBM SPSS Statistics for Macintosh, Version 22.0, Armonk, NY, USA: IBM Corp.), applying a 95% confidence interval (CI) and a significance level of 0.05.

## 3. Results

Two hundred young participants were involved in the study, including 112 boys (56%) and 88 girls (44%). The distribution of study children had a mean age of 7.8 for boys and 8.0 for girls. The mean age was statistically non-significant according to gender.

**Figure 1:**
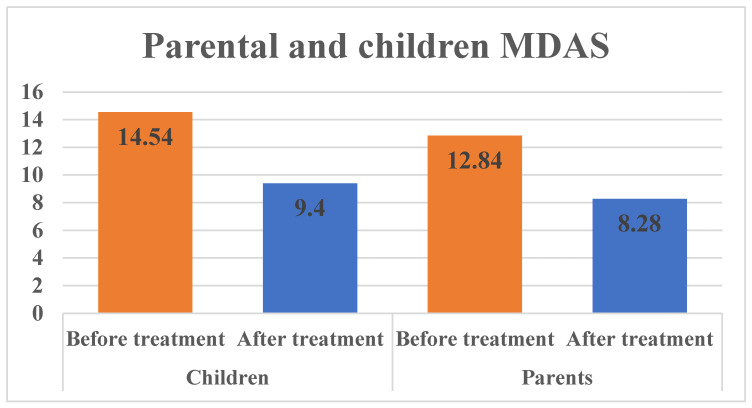
Parental and children modified MDAS scale score.

As shown in Table 2, the perception of children MDAS was found to have the highest mean score of 14.54 ± 3.82 before the dental procedure compared to the mean score of 9.40 ± 2.90 after the dental procedure; this difference was statistically significant (*p* < 0.001). Similarly, for the parents, the perception of MDAS was higher before treatment (12.84 ± 1.50) than after treatment (8.28 ± 2.79). This difference in parental perception was also statistically significant (*p* < 0.001). Figure 1 shows children’s and parents’ modified dental anxiety scores before and after the procedure. In both groups, the dental anxiety score was higher before the procedure than after the procedure.

**Figure 2:**
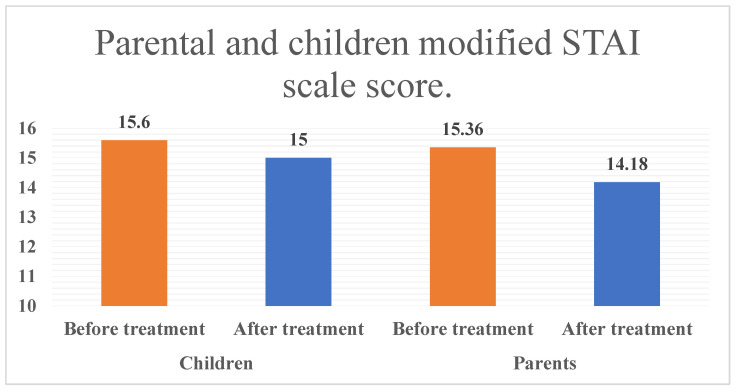
Parental and children modified STAI scale score.

Table 3 shows the modified short STAI scale scores for parents and children. In children, the anxiety rating scores were almost similar before and after treatment. This was statistically non-significant (*p* > 0.05) but highlights a slight reduction in anxiety scores after the procedure. For parents, the comparison before and after the procedure was statistically significant (*p* < 0.05). When comparing the modified STAI scale scores of children and parents before and after the procedure, the dental anxiety score was higher before than after the procedure, as shown in Figure 2. The average anxiety scores of parents based on their education level and socioeconomic status showed no significant differences (refer to Table 4). Nevertheless, an important difference was noted in the MDAS when examining the relationship between the anxiety scores of parents and their children post-dental procedure and in the modified STAI scale before the dental procedure, as indicated in Table 5.

In the larger study sample of 200 children, dental anxiety levels are observed as follows: 76 children experienced “extreme” anxiety, while 26 reported mild anxiety, and 98 had no anxiety, according to the MDAS. The prevalence distribution of emotional responses on the modified STAI scale showed the following: “I feel calm” (46 reported “none”, 41 reported “mild”, 113 reported “extreme”), “I am tense” (166 reported “none”, 25 reported “mild”, 9 reported “extreme”), “I feel upset” (184 reported “none”, 14 reported “mild”, 6 reported “extreme”), “I am relaxed” (31 reported “none”, 46 reported “mild”, 123 reported “extreme”), “I feel content” (33 reported “none”, 33 reported “mild”, 135 reported “extreme”), “I am worried” (145 reported “none”, 37 reported “mild”, 18 reported “extreme”).

Additionally, a weak negative correlation (−0.10) was observed between age and the MDAS total score, indicating that older children tend to report slightly lower levels of dental anxiety.

## 4. Discussion

This research explored dental anxiety in both children and their parents within Al-Kharj, Saudi Arabia. It analyzed anxiety levels using the Modified Dental Anxiety Scale and the modified STAI scale, widely recognized tools for measuring dental anxiety and designed to evaluate psychological stress [20]. A key element of this research was that it took place in a dental office environment instead of a school or other more tranquil setting environment. In the present study, 38% of children were seen with a score higher than 19 on the MDAS before the dental procedure, which is considered severely anxious. This finding surpasses the anxiety levels observed by Kothari et al., who noted that 16.8% of the children in their study were anxious [21]. Dental anxiety arises from multiple factors, and one significant environmental influence is the dental fear exhibited by parents, which closely correlates with that of their children [22]. A mother’s anxiety during childhood may lead to less cooperative behaviors in her children. Additionally, parental anxiety can affect how well a child is mentally prepared for dental procedures, which may hinder the effectiveness of dental care [23]. Prior research indicates that anxiety experienced during dental procedures in children is closely linked to traumatic and negative dental experiences [24,25]. Therefore, this study focused exclusively on children attending their initial dental appointment. Also, in the present study, the type of experience during dental care was not considered to make all the children standardized for the same kind of dental procedure. Our results found no relationship observed between the educational and socioeconomic status of the parent and the anxiety levels. Research indicates that children from families with lower incomes are more prone to experience anxiety during dental visits compared to those from higher-income families. Additionally, it has been noted that dental anxiety is observed more frequently in women than in men [26,27]. Therefore, in this study, we have considered only mothers for proxy evaluation of the anxiety levels of children. When examining the connection between the anxiety levels of parents and their children before and after dental procedures, the findings indicated a positive correlation between the anxiety levels of parents and those of their children at both observed times (*p* < 0.05). The possibility of parents unintentionally passing on their fear of dental procedures to their children in clinical settings is well-established by the results of this study. These findings align with those of Themessl-Huber et al., who conducted a meta-analysis revealing a significant link between parental and child dental anxiety [28]. Additional research also indicates a connection between the anxiety levels of parents and their children [29,30]. Our findings also revealed a notable positive decrease in children’s anxiety levels from before the dental procedure to after it. Therefore, it was observed that children’s anxiety levels diminished over time, consistent with the work of Oppenheim MN et al., who investigated children’s cooperative behavior during dental examinations and subsequent treatment visits, concluding that cooperation improved on the second visit [31]. Furthermore, another study demonstrated that many children experienced anxiety during their initial dental treatment visit [32]. In children, the MDAS score was very high before the dental procedure, possibly due to difficulty rating the scale compared to the modified short STAI (emoji) scale. These findings align with an earlier study in which the researchers observed that all children showed enhanced behavior during later visits [33]. However, the current research provides valuable insights; it does not offer a comparative analysis of parental anxiety levels as a potential indicator of child anxiety. To enhance our understanding of this relationship, further research using a variety of assessment scales would be beneficial. The findings of this study highlight the effectiveness of the modified MDAS and STAI scales in measuring dental anxiety in children. Although no prevalence data were specifically collected, the significant reduction in anxiety scores before and after the procedure supports the hypothesis that dental experience may reduce children’s anxiety levels. Further research could explore the relationship between dental anxiety and demographic factors such as age and gender.

## 5. Conclusions

In the current investigation, we assessed how parental anxiety affects children’s dental fear and examined the levels of children’s dental anxiety both before and following a dental procedure. Consequently, recognizing the anxiety levels of parents who accompany their children can assist clinicians in customizing behavior management techniques for the child. The majority of children displayed a reduction in dental anxiety levels after the dental procedure. As a result, gaining procedural experience can enhance the child’s responses. Consequently, the revised short STAI (emoji) scale serves as a simpler alternative to the MDAS for evaluating anxiety levels in young children.

## Figures and Tables

**Table 1:** Modified Dental Anxiety Scale questions [19] and Modified short STAI (State-Trait Anxiety Inventory).

Sl No	Questions			
1	If you had to go to the dentist for checkup tomorrow, how would you feel?			
2	If you were sitting in the waiting room, how would you feel?			
3	If you were about to have a tooth drilled, how would you feel?			
4	If you were about to have your teeth scaled and polished, how would you feel?			
5	If you were about to receive local anaesthetic injection in your gum, how would you feel?			
* 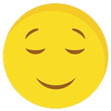 *	I feel calm			
	Not at all = 4	Not at all = 4	Not at all = 4	Not at all = 4
* 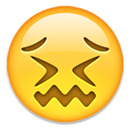 *	I am tense			
	Not at all = 1	Not at all = 1	Not at all = 1	Not at all = 1
* 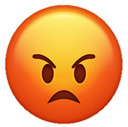 *	I feel sad			
	Not at all = 1	Not at all = 1	Not at all = 1	Not at all = 1
* 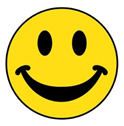 *	I am happy			
	Not at all = 4	Not at all = 4	Not at all = 4	Not at all = 4
* 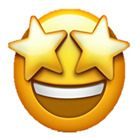 *	I feel excited			
	Not at all = 4	Not at all = 4	Not at all = 4	Not at all = 4
* 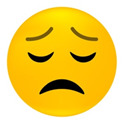 *	I am fearful			
	Not at all = 1	Not at all = 1	Not at all = 1	Not at all = 1

**Table 2:** Evaluation of the Modified Dental Anxiety Scale before and after treatment among children and parents.

		Mean	Std. Deviation	
Children	Before treatment	14.54	3.82	t = 5.75 *p* < 0.001 *
	After treatment	9.40	2.90	
Parents	Before treatment	12.84	1.50	t = 10.49 *p* < 0.001 *
	After treatment	8.28	2.79	
Overall Sample	Mean MDAS	10.33	1.5 (Median)	IQR = 2.0

*: *p* < 0.05.

**Table 3:** The modified STAI scale was evaluated before and after treatment among children and parents.

		Mean	Std. Deviation	
Children	Before treatment	15.60	1.67	t = 1.967 *p* = 0.055
	After treatment	15.00	1.10	
Parents	Before treatment	15.36	1.83	t = 4.65 *p* < 0.001 *
	After treatment	14.18	1.45	

*: *p* < 0.05.

**Table 4:** Comparison of mean anxiety scores of parents according to education and socioeconomic status.

		Mean	Std. Deviation	F Value
Education	Up to diploma	12.61	1.82	1.963
	Bachelors	12.53	1.40	
	Maters	13.50	0.75	
Socioeconomic status	Middle class	12.90	1.41	1.310
	Upper middle	12.44	1.58	
	Upper class	12.33	1.49	

**Table 5:** Correlations between parents and children before treatment.

		Before Treatment	After Treatment
Modified Dental Anxiety Scale	r	0.248	0.580
	Significance	0.082	0.001 *
Modified STAI scale	r	0.424	0.231
	Significance	0.002 *	0.107

*: *p* < 0.05.

## Data Availability

Data sharing is not relevant due to privacy.
